# Changes in soil fungal communities after onset of wheat yellow mosaic virus disease

**DOI:** 10.3389/fbioe.2022.1033991

**Published:** 2022-10-17

**Authors:** Qisen Lu, Cailin Hu, Linna Cai, Chuanfa Wu, Haoqing Zhang, Liang Wei, Tianye Zhang, Haichao Hu, Shuang Liu, Jiajia Lei, Tida Ge, Liangying Dai, Jian Yang, Jianping Chen

**Affiliations:** ^1^ College of Plant Protection, Hunan Agricultural University, Changsha, China; ^2^ State Key Laboratory for Quality and Safety of Agro-products, Institute of Plant Virology, Ningbo University, Ningbo, China

**Keywords:** fungal community, bulk soil, rhizosphere soil, co-occurrence network, plant health

## Abstract

Rhizosphere-associated microbes have important implications for plant health, but knowledge of the association between the pathological conditions of soil-borne virus-infected wheat and soil microbial communities, especially changes in fungal communities, remains limited. We investigated the succession of fungal communities from bulk soil to wheat rhizosphere soil in both infected and healthy plants using amplicon sequencing methods, and assessed their potential role in plant health. The results showed that the diversity of fungi in wheat rhizosphere and bulk soils significantly differed post wheat yellow mosaic virus disease onset. The structure differences in fungal community at the two wheat health states or two compartment niches were evident, soil physicochemical properties (i.e., NH_4_^+^) contribute to differences in fungal community structure and alpha diversity. Comparison analysis showed Mortierellomycetes and Dothideomycetes as dominant communities in healthy wheat soils at class level. The genus Pyronemataceae and *Solicoccozyma* were significantly are significantly enriched in rhizosphere soil of diseased plant, the genus *Cystofilobasidium*, *Cladosporium*, *Mortierella*, and *Stephanonectria* are significantly enriched in bulk soil of healthy plant. Co-occurrence network analysis showed that the fungi in healthy wheat soil has higher mutual benefit and connectivity compared with diseased wheat. The results of this study demonstrated that the occurrence of wheat yellow mosaic virus diseases altered both fungal community diversity and composition, and that NH_4_^+^ is the most important soil physicochemical factor influencing fungal diversity and community composition.

## Highlights

• The soil physicochemical properties of diseased wheat and healthy wheat differed
• The composition of soil fungal community of healthy and diseased wheats differed.
• The co-occurrence networks of soil fungal communities significantly decreased in the soil of diseased wheat.

## Introduction

The diversity, composition, function and ecological relationship of soil microbial community are closely correlated with the occurrence of soil-borne diseases (T. Kühne, 2009; H. Yang et al., 2017). Previous studies have illustrated that the greater community diversity, the greater its resistance to invasion (C.S. Elton, 1958; C. Roscher et al., 2009; R.W. Heckman et al., 2017; C. Shen et al., 2021). The more significant the diversity of the community, the more stable the ecosystem (T.W. Schoener, 1974). The reduction in soil microbial diversity is soil-borne plant diseases (J.D. Van Elsas et al., 2002). These finding indicate the important role of soil microbial diversity in combating plant diseases. Soil physicochemical properties significantly influence soil microbial diversity. For instance, the contents of organic carbon and total nitrogen in wheat rhizosphere soil were positively correlated with wheat yellow mosaic (WYM) (H. Zhang et al., 2022). Increasing organic input can improve soil microbial diversity and stability (Y. Wang et al., 2017). When continuously diluted soil suspensions were inoculated into sterilized soil, the phylogenetic diversity of inoculated soil was significantly reduced, the modularization of functional gene co-occurrence network was simplified, and the stability of soil microbial community was reduced (W. Xun et al., 2021). In the process of plant resistance to pathogen infection, plants themselves also have the recruitment behavior of beneficial microbial communities, which may be active. The enriched microbial communities can enhance soil resistance by interacting with other microbial communities (C. Wu et al., 2021a; C. Wu et al., 2021b). Therefore, we believe that microbial diversity plays a positive role in soil quality and soil inhibition (P. Garbeva et al., 2004).

Fungi comprises different species: different kinds of fungi have different roles in plant growth and development. Some species of fungi are pathogenic to plant, while others are beneficial (J.M. Raaijmakers et al., 2009), and play a key role in nutrient cycling, pest control, and plant community succession in terrestrial ecosystems (Y.-R. Liu et al., 2021). Pathogenic fungi such as *Fusarium pseudograminearum* can infect many crops, including wheat, leading to *Fusarium* crown rot (A. Blum et al., 2019) and *Fusarium graminearum* can cause *Fusarium* head blight (FHB) of wheat (Goswami and Kistler, 2004); In contrast, beneficial fungi such as Trichoderma have strong biological control effects on plants (M. Ghorbanpour et al., 2018). Arbuscular mycorrhizal fungi serve as protective agents against pathogens and virulence stresses for plants (P. Jeffries et al., 2003). In addition, many fungi secrete proteins that inhibit plant viruses. A fungal protease called AsES can induce plant resistance to viruses (M.D.P. Caro et al., 2022). Crude methanolic extracts of *Neosartorya fischeri* and *Penicillium oxalicum* have the activity of inhibiting *Tobacco mosaic virus* (TMV) (S. Shen et al., 2009). Soil fungal communities affected by plant diseases may also be affected. Studies have reported that viral infection of plants may regulate plants and their defense response pathways, thereby affecting other interactions with plant pathogenic fungi and beneficial fungi (Schoelz and Stewart, 2018). The number of *Bacillus subtilis*, *Bacillus licheniformis* and *Bacillus velezensis* colonizing the rhizosphere was higher in healthy cotton rhizosphere than in cotton rhizosphere infected with *Tobacco streak virus* (TSV) (V. S et al., 2018).

WYM is a soil-borne wheat disease mainly caused by *Chinese wheat yellow mosaic virus* (CWMV) in China (Yang et al., 2001). It is a new virus within the genus *Furovirus* and transmitted by an obligate root-infected fungus-like organism, *Polymyxa graminis* (J. Yang et al., 2022). Cases of this virus have been reported in North America, Europe and East Asia and causes different levels of damage to wheat grain yield (T. Kühne, 2009). In severe cases, CWMV causes chlorotic streaking, dwarf symptoms and even death of wheat plants. In wheat producing areas such as Shandong and Henan provinces of China (B.J. Sun et al., 2013). CWMV remains one of the main pathogenic viruses causing mixed infection with *Wheat yellow mosaic virus* (WYMV), thereby reducing wheat yield by an average of 20–30%, but in severe cases, results in no harvest (J.P. Yang et al., 2002). Although wheat yellow mosaic virus disease has caused significant damage to wheat yield, there is currently a lack of effective control methods. The direct and effective control method is the breeding and screening of resistant varieties (J.P. Yang et al., 2002). Effective transgenic varieties have been cultivated in recent years (W. Fu et al., 2016), however, there remain concerns regarding genetic modification affecting the normal wheat metabolism and increase undesirable metabolites in wheat (Kok and Kuiper, 2003).

Due to the important role of soil fungi in plant virus diseases, we attempted to explore and describe the effects of wheat yellow mosaic virus disease on soil fungal community, to provide evidence for the biological control of wheat yellow mosaic virus disease. We propose the following hypotheses: 1) the occurrence of wheat yellow mosaic virus disease can affect the composition of soil fungal community, soil physicochemical properties, and the associations between fungal communities; 2) the enrichment variations of the fungal communities in soil were correlated with the occurrence of wheat yellow mosaic virus disease. To test these hypotheses, this study 1) investigated the differences in fungal community composition, soil physicochemical properties, and fungal community between healthy and diseased wheat soils; 2) assessed, *via* co-occurrence network the differences in fungal enrichment communities between healthy and diseased wheat soils.

## Materials and methods

### Sampling sites and growing conditions

Soil samples were collected in Banquan town (35°11′N, 118°64′E), Junan, Shandong, China which has a temperate monsoon climate with an annual average temperature of 25.5°C (http://www.junan.gov.cn/xq/jngk/dlhj.htm). The climate is humid, and the field soil is aquic brown soil. The sampling plots were sown in winter wheat/summer maize rotation system for nine consecutive years (2012–2021), and the wheat variety was Lin Mai 4. Before sowing, the soil was turned over and plowed deeply so that the overall structure and physiochemical properties of the soil were similar, and the cultivation management was consistent with the local cultivation management.

### Sample collection

Wheat yellow mosaic virus disease occurred in wheat returning-green stage. The health status of wheat was assessed and classified before sampling. Healthy plant samples were wheat plants with no symptom, while diseased plant samples were wheat plants with yellowing, shrinkage of leaves, or stunted whole plants. The rhizosphere soil and bulk soil of the diseased and healthy wheat were collected evenly on the diagonal of each plot, and the sampling sites were about 3 m apart. Rhizosphere soil refers to soil particles attached to the root system and can be obtained by strong shaking of the root system Collected soil samples were sealed in valve bag and labeled (A. Lombini et al., 2003). The bulk soil was collected from the soil between ridges where there was no wheat root distribution, sealed, and labeled with self-sealing bags. All samples were stored in low-temperature iceboxes and transported to the laboratory. In a follow-up test, all grouped soil samples were screened (<2 mm) and mixed evenly. Rhizosphere soil and bulk soil were divided into two parts one for DNA extraction and sequencing, and the other for dry rhizosphere soil to determine its chemical properties. The contents of ammonium nitrogen (NH_4_^+^) and nitrate nitrogen (NO_3_^−^) in fresh soil were determined. The sample name: BSD, the bulk soil of the diseased plant; BSH, the bulk soil of the healthy plant; RSD, the rhizosphere soil of disease plants; RSH, the rhizosphere soil of healthy plants.

### Soil physiochemical properties analysis

The soil samples should be treated with 1% HNO_3_ solution and digested with HF to determine the contents of total potassium (TK), total sulfur (S), total copper (Cu), total calcium (Ca), total magnesium (Mg), total iron (Fe), total zinc (Zn), total boron (B) and molybdenum (Mo) (C. Wu et al., 2021b; H. Zhang et al., 2021) Atomic absorption spectrometer (Novaa350, Jena, Germany) was then used to determine the content of TK, and the contents of the other elements were measured using an inductively coupled plasma spectrometer (ICP-OS-5110, Agilent, America). Soil available phosphorus (AP) and soil total phosphorus (TP) contents were extracted with NaHCO_3_ and measured using a UV-Vis spectrophotometer (UV-2600, Shimadzu, Japan) (C. Wu et al., 2021b). The soil organic carbon (SOC) content was calculated using the K_2_Cr_2_O_7_ volumetric method. The soil total nitrogen (TN) was extracted with sulfuric acid and then measured using a Vario Max Element Analyzer (Vario Max, Elementar, Germany) (Z. Zhang et al., 2017). After extraction with 1 M KCl solution, NH_4_^+^ and NO_3_^−^ were measured using a continuous flow analysis system (AA3, Seal Analytical, Germany) (Y. Shi et al., 2018).

### DNA extraction, PCR assays, high-throughput sequencing

A 0.5 g of rhizosphere soil and bulk soil from five duplicated wheat samples were used for DNA extraction. Rhizosphere soil DNA and bulk soil DNA were extracted using the DNeasy Power Soil Kit and detailed steps were referred to the built-in instructions. The concentration of extracted DNA was detected by Nanodrop RND-2000 (NanoDrop Technologies, Wilmington, DE, United States).

The internal transcribed spacer 1 (ITS1) region of fungi were amplified with the primer pair ITS5-1737F (5′-GGA​AGT​AAA​AGT​CGT​AAC​AAG​G-3′) and ITS2-2043R (5′-GCT​GCG​TTC​TTC​ATC​GAT​GC-3′). PCR reaction consisted of 12.5 μL 2 × Premix Taq™ (TaKaRa. Bio Inc. Shiga, Japan), 1 μL of each primer (10 μm), 2 μL of DNA extract (5–20 ng), and 9 μL of ddH_2_O to a final volume of 25 µL. PCR amplification was sequenced as follows: desaturated at 95°C for 5 min; followed by 35 cycles at 94°C, 50 s at 58°C and 30 s at 68°C and a final extension step at 68°C for 10 min. MiSeq sequences of purified amplicons were high-throughput sequenced by Novogene Biopharm Technology Co. Ltd (Tianjin, China) using Illumina^®^ MiSeq sequencer (Illumina, San Diego, CA, United States).

The DNA sequencing data were uploaded to the National Center for Biotechnology Information (NCBI) Sequence Read Archive (SRA) database under accession number SRR 6008834.

### Bioinformatics analysis

Fast Length Adjustment of Short reads was used to merge the DNA sequences obtained by sequencing (Magoč and Salzberg, 2011) and the merged sequences were processed with QIIME2 (E. Bolyen et al., 2019). According to the method of Callahan et al., the obtained original sequences were demultiplexed and quality filtered with Q2-demux, then denoised with DADA2 (B.J. Callahan et al., 2016). According to the methods of Nakamura et al. ‘s and Price et al., amplicon sequence variants (ASVs) were compared with MAFFT and inducted phylogenetic with FastTree 2 (M.N. Price et al., 2009; T. Nakamura et al., 2018). Fungal identifications were compared in the UNITE database (R.H. Nilsson et al., 2019). Before the community analysis, we deleted the OTU sequence less than two reads (E.M. Bach et al., 2018), and standardized the read depth (19,279 reads per sample) to ensure the reliability of the data.

### Statistical analysis

Duncan’s test in IBM SPSS Statistics 19 was used to analyze the differences in soil physicochemical properties among the treatments (M.-Y. Zheng et al., 2021) after one-way variance analysis (ANOVA) to detected significance. Based on ASVs data, microbial diversity analysis was performed on the sampled plots. Diversity indices including Shannon diversity index, Richness index, Simpson index and ACE index were calculated using the “vegan” R package (https://cran.r-project.org/). Principal co-ordinates analysis (PCoA) was carried out based on the Bray–Curtis distance matrices to assess beta diversity of fungal communities, analysis of similarities (ANOSIM) tests were used for testing significant differences. The Mantel test was used to evaluate the relationship between soil fungal communities and soil physicochemical properties (S. Sunagawa et al., 2015). In order to determine the most important predictors of disease index, a randomForest model was built on R platform (4.0.2) using the “randomForest” R package (Liaw and Wiener, 2001). The importance of the model and each predictor was assessed using the “A3” and “rfPermute” R packages, respectively (M. Delgado-Baquerizo et al., 2020).

STAMP software was used to compare soil fungal community composition under different plant states, The differences were tested using Welch’s *t*-test (*p* < 0.05) (Parks et al., 2014). To maintain stability (Spearman’s R > 0.8 or R < -0.8) and statistically significant (*p* < 0.01) correlation, the “Psych” R package was used to conduct species-level symbiotic network analysis of fungal communities on the R platform. To avoid possible bias, more than 60% of the genera present in the sample were reserved for network analysis (C. Wu et al., 2021b). The completed Co-occurrence network relationship was visualized graphically in Gephi. The obtained analysis results were visualized by Gephi0.9.2 software (P. Du et al., 2021). We determined the within-module connectivity (*Zi*) and among-module connectivity (*Pi*) of the nodes. The nodes are classified using *Zi* and *Pi* according to their topological role in the network. Node topologies are classified as peripherals (Zi < 2.5 and Pi < 0.62), connector (Pi > 0.62), network hub (Zi > 2.5 and Pi > 0.62), and module hubs (*Zi* > 2.5) (Y. Deng et al., 2012), the “ggplot2” R package (*ZiPi* plots) was used for drawing (H. Wickham, 2011).

## Results

### Soil physicochemical properties

In healthy plants soil, the contents of pH, NH_4_^+^, TN, S, Ca, Mg, and Fe in rhizosphere soil were significantly higher than that of bulk soil, and the contents of NO_3_^−^, AP, SOC, TP, TK, Cu, Zn, B, and Mo had no significant difference. In diseased plants soil, the contents of NO3^-^, TP, Cu, and Zn in rhizosphere soil was significantly lower than that in bulk soil, and the contents of NH_4_^+^, TN, and S were significantly higher than that of bulk soil. Notably, we found that the contents of SOC, TN, S, Cu, and Zn in diseased plants were significantly higher in both rhizosphere and bulk soils than in healthy plants. In the bulk soil, the contents of NO_3_^−^, NH_4_^+^, AP, SOC, TN, TP, Cu, Ca, Fe, Zn, and pH in diseased soil were significantly higher than those of healthy soil (*p* < 0.05, Duncan’s test), while the contents of TK, Mg, B and Mo showed no observably differences. In rhizosphere soil, the contents of AP, SOC, TN, S, Cu and Zn in diseased soil was significantly higher than those in healthy soil (*p* < 0.05, Duncan’s test, Table 1), while the content of NO_3_^−^, NH_4_^+^, Ca, Mg, Fe in the healthy soil was significantly higher than that in those diseased soil (*p* < 0.05, Duncan’s test, Table 1), and there were no significant differences in the contents of TP, TK, B, Bo and pH. The contents of NO_3_^−^, TP, Cu, and Zn in the bulk soil of diseased plants were signally higher than those of the other three types of soil (*p* < 0.05, Duncan’s test, Table 1). The contents of NH_4_^+^, Ca, Mg, and Fe in the rhizosphere soil of healthy plants were significantly higher than those of the other three types of soil (*p* < 0.05, Duncan’s test, Table 1).

**TABLE 1:** Physicochemical properties of the bulk soil of healthy plants, the bulk soil of the diseased plants, the rhizosphere soil of the diseased plants and the rhizosphere soil of healthy plants. BSH, the bulk soil of healthy plants. BSD, the bulk soil of the diseased plants. RSH, the rhizosphere soil of healthy plants. RSD, different letters show significant difference among treatments. Values are means ± SEs (Duncan test, *p* < 0.05, *n* = 5). The letters in the table represent different significances (a, b, c). AP = available phosphorus, SOC = soil organic carbon, TN = total nitrogen, TP = total phosphorus, TK = total potassium, S = total sulfur, Cu = total copper, Ca = total calcium, Mg=total magnesium, Fe = total iron, Zn = total zinc, B = total boron, Mo = total molybdenum.

Treatment	BSH	BSD	RSH	RSD
pH	5.41±0.20b	5.83±0.12a	6.03±0.46a	6.48±0.18a
NO_3_^−^ (mg/kg)	20.37±4.88b	34.77±4.64a	18.76±5.78b	8.29±3.61c
NH_4_^+^ (mg/kg)	8.48±3.35d	44.16±23.39c	107.3±21.40a	75.39±19.93b
AP (mg/kg)	10.71±3.01b	28.88±1.38a	10.64±2.10b	30.34±8.99a
SOC (g/kg)	7.40±1.26b	15.25±2.28a	8.08±1.11b	15.95±1.41a
TN (g/kg)	0.81±0.09d	1.35±0.18b	1.13±0.06c	1.81±0.11a
TP (g/kg)	0.15±0.03b	0.60±0.23a	0.18±0.03b	0.22±0.01b
TK (g/kg)	11.20±1.03a	13.02±2.228a	12.11±0.74a	12.27±0.27a
S (g/kg)	0.43±0.08c	1.04±0.30b	0.93±0.19b	2.06±0.54a
Cu (g/kg)	0.22±0.01c	0.36±0.01a	0.23±0.02c	0.31±0.04b
Ca (g/kg)	1.87±0.28c	5.65±0.22b	6.94±0.29a	5.96±0.76b
Mg (g/kg)	3.65±0.23b	3.48±0.36b	4.32±0.35a	3.64±0.22b
Fe (g/kg)	18.71±0.65c	20.22±0.58b	22.12±0.95a	19.43±0.76bc
Zn (mg/kg)	42.96±1.43c	75.64±4.13a	45.92±1.51c	65.40±3.58b
B (mg/kg)	11.31±0.52a	13.51±2.29a	12.80±0.37a	13.16±2.22a
Mo (mg/kg)	0.69±0.11b	0.87±0.12ab	0.76±0.15ab	1.00±023a

### Analysis of fungal diversity

**FIGURE 1:**
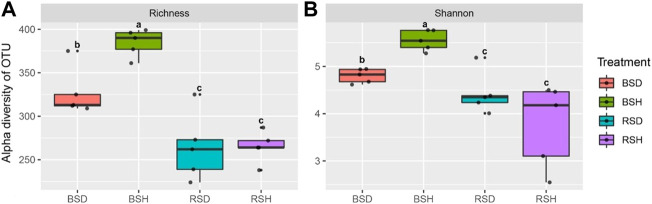
Difference analysis of alpha and beta diversity of fungi among the bulk soil of the diseased plant, the bulk soil of healthy plants, the rhizosphere soil of disease plants and the rhizosphere soil of healthy plants. **(A)** Richness index of BSD, BSH, RSD, and RSH; **(B)** Shannon index of BSD, BSH, RSD, and RSH; BSD, the bulk soil of the diseased plant; BSH, the bulk soil of healthy plants; RSD, the rhizosphere soil of disease plants; RSH, the rhizosphere soil of healthy plants. Significance satisfies *p* < 0.05 based on Tukey test.

**FIGURE 2:**
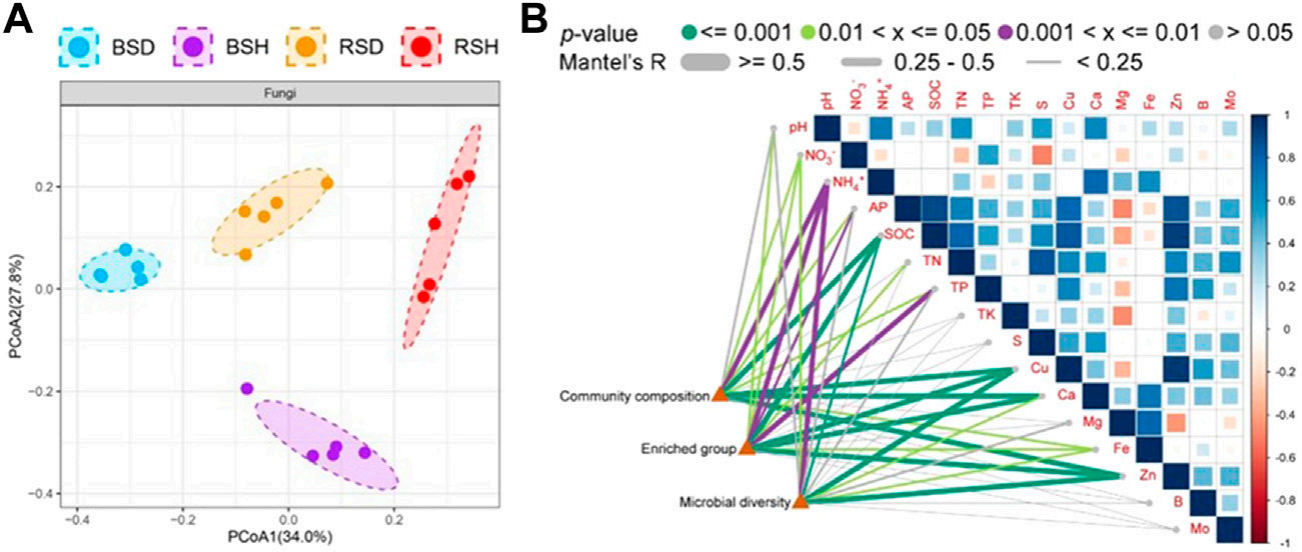
Difference analysis of beta diversity of fungi among the bulk soil of the diseased plant, the bulk soil of healthy plants, the rhizosphere soil of disease plants and the rhizosphere soil of healthy plants and pairwise correlation analysis between environmental factors and community composition, microbial diversity and enriched group **(A)** Principal co-ordinates analysis (PCoA) indicated the beta diversity of fungi community. Significance satisfies *p* < 0.05 based on Tukey test. BSD, the bulk soil of the diseased plant; BSH, the bulk soil of healthy plants; RSD, the rhizosphere soil of disease plants; RSH, the rhizosphere soil of healthy plants. **(B)** Different colors represent different ranges of *p*-values and different degrees of significance, Significance satisfies *p* < 0.05 based on Tukey test. Different line thicknesses represent different R values obtained through Mantel detection, and represent different correlation degrees. Microbial diversity was analyzed at the level of species, Enriched group was analyzed at the level of genus, and community composition was analyzed at the level of family.

We investigated the alpha diversity of four different soil fungal communities. The results showed that the differences of the richness index of the four soil fungal communities were the same as the differences of Shannon index (Figure 1). The richness index and Shannon index of fungal communities in BSH treatment were significantly higher than those in the other three soils (*p* < 0.05, Tukey test), followed by BSD treatment, but there was no significant difference in richness index and Shannon index between RSD and RSH treatments. The results also showed that the alpha diversity of soil fungal community in diseased wheat significantly decreased compared with that in bulk soil, while the alpha diversity of rhizosphere soil fungal community did not significantly change. We performed principal co-ordinate analysis (PCoA) and found differences in fungal community structure among different soil compartments (Figure 2A). Correlation analysis showed NH_4_^+^ was the most important physicochemical factors affecting fungal diversity and community composition (Figure 2B).

### Fugal community composition

**FIGURE 3:**
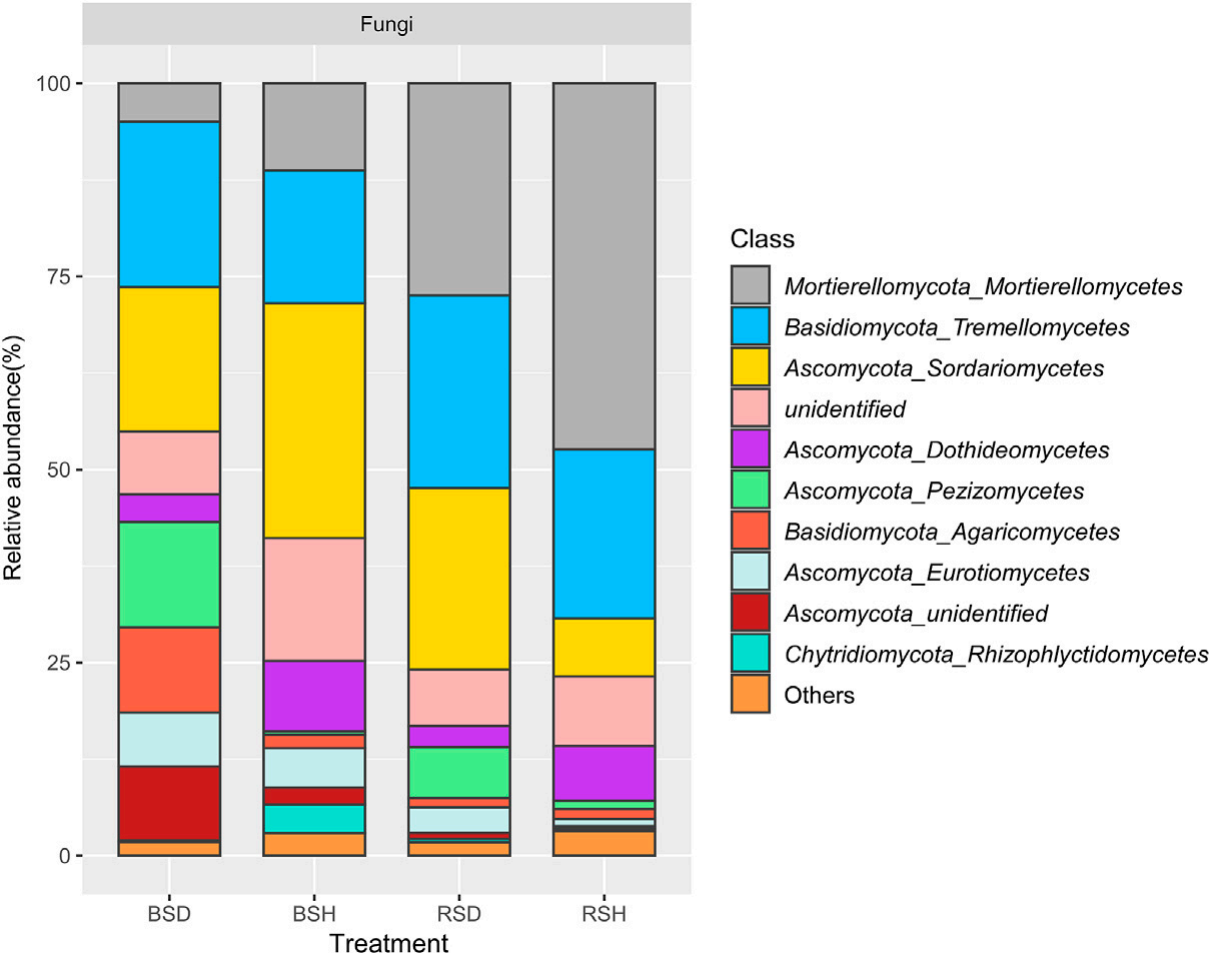
Relative abundance of 10 dominant classes in fungi among the bulk soil of the diseased plant, the bulk soil of healthy plants, the rhizosphere soil of disease plants and the rhizosphere soil of healthy plants. Family accounting for less than 1% of total reads were classified as “others”. BSD, the bulk soil of the diseased plant; BSH, the bulk soil of healthy plants; RSD, the rhizosphere soil of disease plants; RSH, the rhizosphere soil of healthy plants Significance satisfies *p* < 0.05 based on Tukey test.

To further explore the differences in soil fungal community structure between healthy and diseased plants, we analyzed the fungal community composition. The results showed the proportion of *Mortierellomycetes* and *Dothideomycetes* in the healthy treatment group was higher than that in the disease treatment group (Figure 3; Supplementary Table S1). *Tremellomycetes* (21.43%), *Sordariomycetes* (18.71%) and *Pezizomycetes* (13.65%) were the dominant in BSD treatment; *Sordariomycetes* (30.38%), *Tremellomycetes* (17.22%) and *Mortierellomycetes* (11.29%) were the dominant in BSH. In addition, the proportion of *Mortierellomycetes* and *Sordariomycetes* in BSH treatment was higher than that in BSD treatment (Figure 3; Supplementary Table S1). In the rhizosphere soil, *Mortierellomycetes* (27.44%), *Tremellomycetes* (24.95%) and *Sordariomycetes* (23.54%) were the dominant classes in RSD treatment; *Mortierellomycetes* (47.39%), *Tremellomycetes* (21.91%) and *Sordariomycetes* (7.48%) were the dominant classes in RSH treatment (Supplementary Table S1). Comparative analysis revealed that *Fusarium*, Pyronemataceae and *Solicoccozyma* were significantly enriched in RSD treatment (Supplementary Figure S1A) and P. *Pseudaleur* was significantly enriched in the BSD treatment (Supplementary Figure S1B). *Cystofilobasidium*, *Cladosporium*, *Mortierella*, *Stephanonectria* were significantly enriched in BSH treatment (Supplementary Figure S1B). In conclusion, fungal community structure and composition in rhizosphere and bulk soils of wheat yellow mosaic virus disease were changed compared with those of healthy plants.

### Network association in different treatments

**FIGURE 4:**
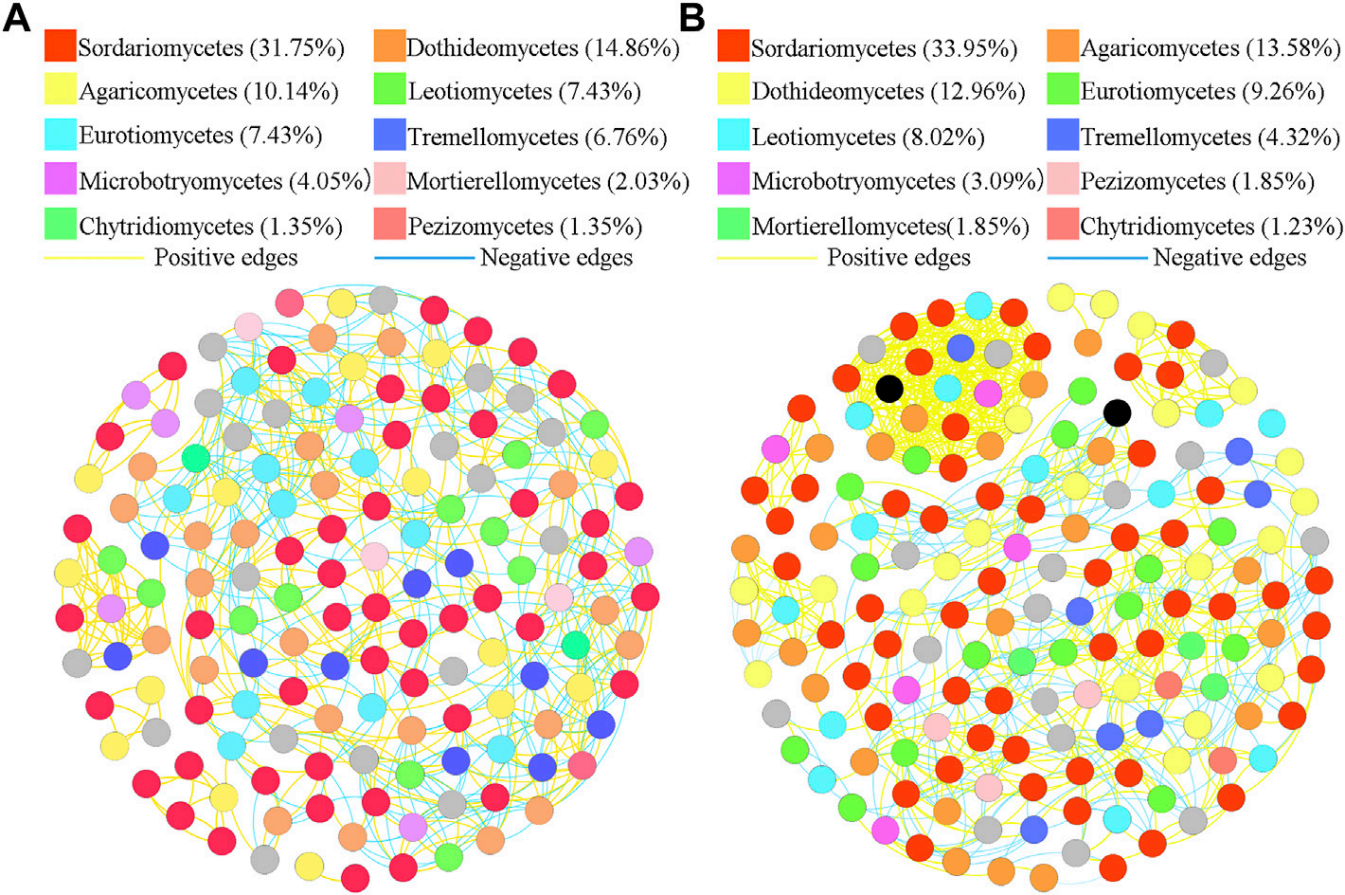
Schematic representation of symbiotic network patterns of soil fungi under different treatments at the genus level. These dots represented different genera of fungi that were clustered at class level. Different colors of the squares represent different dominant fungal genera that were clustered (only list the top ten fungal classes). Yellow lines between any connected two nodes indicates a positive relationship and blue lines indicate a negative relationship **(A)** Schematic diagram of fungal community network in healthy bulk soils. **(B)** Schematic diagram of fungal community network in diseased bulk soils.

**FIGURE 5:**
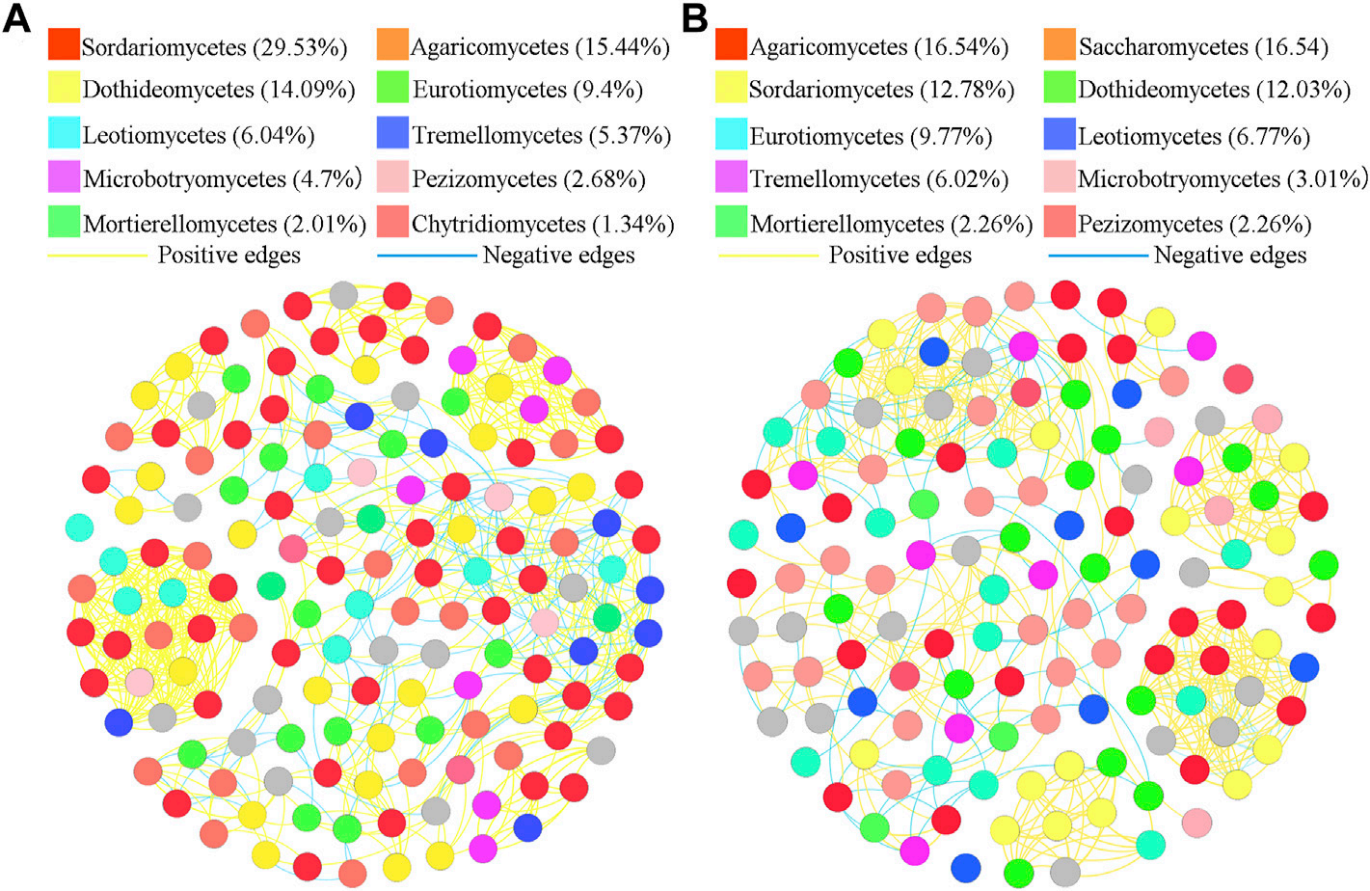
Schematic representation of symbiotic network patterns of soil fungi under different treatments at the genus level. These dots represented different genera of fungi that were clustered at class level. Different colors of the squares represent different dominant fungal genera that were clustered (only list the top ten fungal classes). Yellow lines between any connected two nodes indicates a positive relationship and blue lines indicate a negative relationship **(A)** Schematic diagram of fungal community network in healthy rhizosphere soils. **(B)** Schematic diagram of fungal community network in diseased rhizosphere soils.

To better understand the interrelationships among different fungal communities in the four treatments, we generated fungal networks to describe the symbiotic patterns among fungal communities at the genus level (Figures 4, 5). In these bulk soils, the network nodes were mainly divided into *Sordariomycetes*, *Dothideomycetes*, *Agaricomycetes*, *Leotiomycetes*, *Eurotiomycetes*, *Tremellomycetes*, *Microbotryomycetes*, *Mortierellomycetes*, *Pezizomycetes*, *Chytridiomycetes* (Figure 4). The proportions of *Sordariomycetes* (31.75%), *Dothideomycetes* (14.85%), and *Agaricomycetes* (10.14%) (Figure 4A) were the three classes with the largest proportions in healthy plant soils were different from that of *Sordariomycetes* (33.95%), and Agaricomycetes (13.58%) (Figure 4B) in the soil of diseased plants. The analysis of network topological properties revealed that the soil fungal community network of BSH treatment was simpler than that of BSD treatment, and the constructed BSH fungal network contained 148 nodes, 608 links, with an average connectivity of 0.66, and a modularity of 2.43 while the BSD network had 164 nodes, 777 links, with an average connectivity of 0.77, and modularity of 0.80 (Table 2). In these rhizosphere soils, the RSH network nodes were mainly divided into *Sordariomycetes*, *Agaricomycetes*, *Dothideomycetes*, *Eurotiomycetes*, *Leotiomycetes*, *Tremellomycetes*, *Microbotryomycetes*, *Pezizomycetes*, *Mortierellomycetes*, *Chytridiomycetes*, while the RSD network nodes were mainly divided into *Agaricomycetes*, *Saccharomycetes*, *Sordariomycetes*, *Dothideomycetes*, *Eurotiomycetes*, *Leotiomycetes*, *Tremellomycetes*, *Microbotryomycetes*, *Mortierellomycetes*, *Pezizomycetes* (Figure 5). The top four classes of the RSH treatment included four keystone taxa, *Sordariomycetes* (29.53%), *Agaricomycetes* (15.44%), *Dothideomycetes* (14.09%), *Eurotiomycetes* (9.4%) (Figure 5A), while the top four classes of the RSD treatment included four keystone taxa, *Agaricomycetes* (16.54%), *Saccharomycetes* (16.54%), *Sordariomycetes* (12.78%), *Dothideomycetes* (12.03%) (Figure 5B). However, when we compared the co-occurrence networks of RSH and RSD treatments, we found that the network nodes were decreased from 149 in RSH treatment to 133 in RSD treatment (Table 2), the network edges were decreased from 678 in RSH treatment to 521 in RSH treatment (Table 2), but the network positive edges were increased from 82.57% in RSH treatment to 85.91% in RSD treatment (Table 2). These network topology parameters indicated that the bulk soil fungal community network became larger, more nodes and more complex after the occurrence of disease. Although the number of nodes and edges of the rhizosphere soil fungal community decreased after the occurrence of the disease, the mutually beneficial relationship among the communities within the network was enhanced.

**TABLE 2:** Microbial co-occurrence network indices. BSD, the bulk soil of the diseased plants. RSH, the rhizosphere soil of healthy plants. RSD, different letters show significant difference among treatments.

Network	BSH	BSD	RSH	RSD
Nodes	148	164	149	133
Edges	608	777	678	521
Positive edge (%)	64.02	75.11	82.57	85.91
Negative edge (%)	35.98	24.89	17.43	14.09
Average degree	10.22	11.46	11.27	7.84
Modularity	2.43	0.80	0.89	0.95
Average clustering coefficient	0.66	0.77	0.79	0.73

**FIGURE 6:**
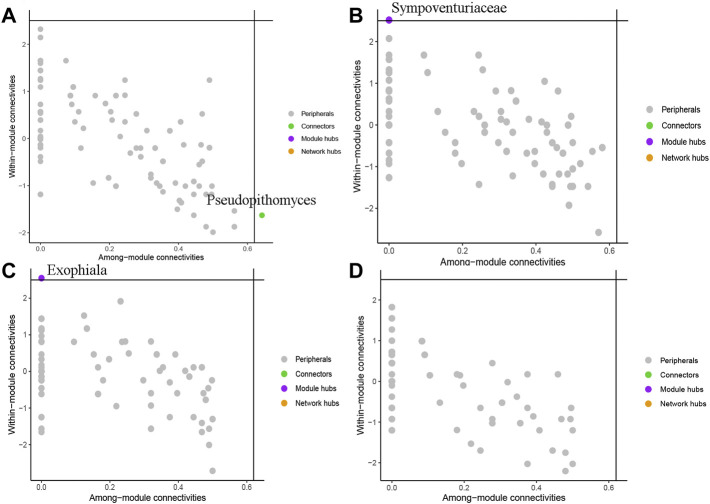
Soil fungi under different treatments were divided into network nodes by within-module connectivity (Zi) among-module connectivity (Pi) **(A)** the bulk soil of healthy plants (BSH) **(B)** the bulk soil of the diseased plant (BSD) **(C)** the rhizosphere soil of healthy plants (RSH) **(D)** the rhizosphere soil of disease plants (RSD).

To evaluate the potential topological role of groups in soil fungal symbiotic networks, nodes were divided into four types (peripherals, connectors, modular hubs and network hubs) according to their within-module connectivity (Zi) and among-module connectivity (Pi) (Figure 6) values. The results show that the genus *Pseudopithomyces* belonged to connectors in BSH treatment (Figure 6A), the family Sympoventuriaceae belonged to Module hubs in BSD treatment (Figure 6B), the genus *Exophiala* belonged to Module hubs in RSH treatment (Figure 6C), and all nodes were classified as peripheral nodes in RSD treatment (Figure 6D). They were all highly connected to other important nodes.

## Discussion

Previous reports have suggested NH_4_^+^ as one of the main factors causing the difference of fungal community among different land use types and plant species in the eastern mountainous area of Liaoning province (Q. Zhang et al., 2021), Arbuscular mycorrhizal fungi were positively correlated with NH_4_^+^ in degraded alpine grassland soil system of Qinghai-Tibet Plateau (J. Li et al., 2020b). NH_4_^+^ was also a major factor affecting the diversity and composition of fungal communities in our study (Figure 2B). This conclusion was consistent with findings of Smithwick et al. and Chen et al. that ammonium nitrogen can significantly affect soil microbial community (E.A.H. Smithwick et al., 2005; W. Chen et al., 2017). In our study, the proportion of NH_4_^+^ in rhizosphere soil was higher than that in bulk soil, which was consistent with previous (Table 1) (M. Koranda et al., 2011; Y. Cui et al., 2021; X. Zhu et al., 2021). This may be attributed to the fact that plant roots stimulate the production and fixation of NH_4_^+^, but limit the production of NO_3_^−^ to meet the needs of plants for NH_4_^+^ (X. Zhu et al., 2021). In this study, the alpha diversity in bulk soil was significantly higher than that in rhizosphere soil, and NH_4_^+^ was closely related to the diversity and community composition of fungal communities, which may be due to the different responses of fungal communities in soils with different treatments to NH_4_^+^, resulting in the growth differences among fungal communities (Y.-C. Bai et al., 2020).

Through analysis of relative abundance, our results showed that Ascomycota and Basidiomycota were the two most common fungal phyla in the four soils (Figure 3), which is consistent with the results of previous studies (X.-g. Li et al., 2014b; J. Jiang et al., 2019). We observed changes in community composition between treatments at the class level. Notably, compared with healthy wheat, both in rhizosphere soil and bulk soil, the proportion of *Mortierellomycetes* and *Dothideomycetes* in healthy wheat was higher than that in diseased wheat (Supplementary Table S1). These two classes of fungi exist in a variety of soils with resistance to pathogen infection (X. Liu et al., 2021; T. Xu et al., 2021). Many genera of the class *Mortierellomycetes* belong to plant growth-promoting fungi (PGPF), which play a positive role in plant resistance to pathogen invasion (Ozimek and Hanaka, 2021). The class *Dothideomycetes* are the largest class of fungi, many of which are important for plant resistance to disease and stress (D. Pem et al., 2021). *Cystofilobasidium*, *Cladosporium*, *Mortierella* and *Stephanonectria* attracted our attention for their significant enrichment in BSH treatment. These genera have been reported to produce positive responses to plant growth or soil resistance by themselves in the soil. For instance, the genus *Cystofilobasidium* usually exists as the dominant community in compost and humus (J. Li et al., 2020a). The genus *Cladosporium* is a widely distributed endophytic fungus that protects plants from biological and abiotic stresses. These species secrete beneficial secondary metabolites that improve the ability of plants to adapt to new habitats and maintain plant health and performance (Achard and Genschik, 2009; Răut et al., 2021). The genus *Mortierella* helps crops and mycorrhizal fungi capture phosphorus (P) as well as decompose plant litter and degrade aromatics (L. Ellegaard-Jensen et al., 2013; Tamayo-Velez and Osorio, 2016). When investigating fungal enrichment communities in different soils, we found that the genera *Fusarium* was significantly enriched in RSD treatment, this phenomenon has been found in other plants as well (M. Guan et al., 2018; H. Liu et al., 2019). We determined that the genus *Fusarium* was significantly enriched in RSD treatment as a common pathogenic fungus (Steinkellner and Langer, 2004). The enrichment of *Fusarium* may lead to the occurrence of wheat root diseases and damage of wheat root surface (M. Winter et al., 2019), which may be more conducive to the parasitism of *P. graminis* on wheat roots (K. Hansen et al., 2013; Mestre and Fontenla, 2021). In conclusion, the occurrence of Wheat yellow mosaic virus disease affects the composition of soil fungal community, but there are many reasons for this change, such as soil physicochemical interaction and soil nutrient imbalance (C.H. Kong et al., 2008; C. Li et al., 2010), may also be due to the promoting or inhibiting effect of plant rhizosphere exudates on the growth of different fungal communities (X.-g. Li et al., 2014a), more detailed reasons need to be explored.

In recent years, network co-occurrence analysis has provided a reliable method to study the symbiotic mode and mutual relationship of soil microbial communities in complex environments (Faust and Raes, 2012). Previous reports have confirmed that the interaction and connectivity between communities affect the stability and persistence of communities, and the network structure of these interactions is correlated with the community invasion resistance (Thébault and Fontaine, 2010; Xu et al., 2020; Ji et al., 2021). We found that the soil fungal community network of BSH treatment was simpler than that of BDH treatment, it may be that the contents of NO_3_^−^ and NH_4_^+^ in the rhizosphere soil is significantly higher than healthy wheat soil (Table 1), thus resulting in a loss of some fungal community richness (L.C. Cline et al., 2018; S. Jiang et al., 2018). In rhizosphere soil, RSH treatment had more edges and nodes than the RSD treatment, suggesting that the soil fungal network of healthy plants was more larger. Complex fungal networks in the soil lead to better community stability and provide better anti-interference (W. Yang et al., 2019; L. Xu et al., 2020) Simultaneously, a higher positive correlation edge in soil fungal communities of healthy plants may mean more cooperation (J. Zhang et al., 2021) and a greater connected network provides more functional redundancy (Mougi and Kondoh, 2012). In addition, more complex networks can cope with diverse and complex environmental changes or inhibit infection of plants by soil-transmitted pathogens (H. Yang et al., 2017; J. Tao et al., 2018). Highly diverse microbial communities have a better ability to resist pathogen infection, which makes the soil itself more resistant (C.A. Mallon et al., 2015). The mutualistic relationship between fungal communities in diseased wheat was broken, and the complexity and stability of the community were reduced, which may provide a more favorable environment for pathogen infection (N.L. Nwokolo et al., 2021).

Due to the importance of dominant groups, biomarker groups and network hub groups in ecology and evolutionary biology, many studies of microorganisms are aimed at finding and identifying them in networks (S. Banerjee et al., 2018; C. Xiong et al., 2021). We found two key genera and one family (*Pseudopithomyces*, *Exophial*, and Sympoventuriaceae) in different treatments. There are many species in the family *Pseudopithomyces*, including both pathogenic fungi and fungi that inhibit pathogens (A. Perelló et al., 2017; B.N.S. Ningsih et al., 2021). The family Sympoventuriaceae is a family of saprophytic and pathogenic fungi (M. Shen et al., 2020). Most species of *Exophiala* can cause plant diseases and human diseases (E.J. Otis et al., 1985; De Hoog et al., 2005). This study demonstrates the critical role of these fungal communities in the fungal community network after the onset of wheat yellow mosaic virus disease. Our results suggest that these fungal communities play an important role in the fungal community network after the occurrence of wheat yellow mosaic virus disease, so as to better understand the changes of fungal communities after the occurrence of wheat yellow mosaic virus disease and the role of these changes in the plant defense response.

In recent years, biological control through biological control agents has been recognized as a promising method to reduce the incidence of many field crop diseases (S. Munir et al., 2022). For example, *Neorhizobium galegae*, *Lysobacter dokdonensis*, and *Ensifer adhaerens* can prey on bacteria and can be used to control Tobacco bacterial wilt (W. Ahmed et al., 2022); *P. camelliae-sinensis* infection can be used to prevent and treat tea gray blight disease (Q. Wang et al., 2021). Our studies identified dominant fungal communities in healthy and diseased plant soils respectively, and we suggest that core communities in healthy plant soils help promote WYM resistance. These identified fungal communities can be developed into biological control agents for WYM control in the future. This will provide a theoretical basis for fungal microbial resistance to WYM to improve wheat yield and a reference community for biological control.

## Data Availability

Publicly available datasets were analyzed in this study. This data can be found here: https://www.ncbi.nlm.nih.gov/sra/?term=. Accession Number: 6008834.
