# Supplementary material for: Changes in soil fungal communities after onset of wheat yellow mosaic virus disease
Source: Front Bioeng Biotechnol. 2022 Oct 17;10:1033991. doi: 10.3389/fbioe.2022.1033991 (PMC9621598; doi:10.3389/fbioe.2022.1033991)
Supplement: Supplementary file 1 [file Table1.DOCX]

Table S1 The relative abundance of dominant funge class among different treatment. BSH, the bulk soil of healthy plants. RSH, the rhizosphere soil of healthy plants. BSD, the bulk soil of the diseased plants. RSD, the rhizosphere soil of the diseased plants. Values are means ± SEs (Tukey test, *p* < 0.05, n = 5). Different letters show significant difference among treatments (a, b, c).

| **Class** | | **BSH** | **RSH** | **BSD** | **RSD** |
| --- | --- | --- | --- | --- | --- |
| *Mortierellomycetes* | 11.285±1.767bc | | 47.395±20.848a | 4.949±2.450c | 27.435±5.223ab |
| *Tremellomycetes* | | 17.219±3.984a | 21.914±12.161a | 21.427±6.595a | 24.95±3.344a |
| *Sordariomycetes* | | 30.378±4.385a | 7.483±1.678c | 18.710±1.355b | 23.543±2.806b |
| Fungi_unidentified | | 15.912±2.219a | 8.984±3.462b | 8.131±1.746b | 7.2739±1.770b |
| *Dothideomycetes* | | 9.101±2.666a | 7.119±3.619ab | 3.591±0.042b | 2.754±1.243b |
| *Pezizomycetes* | | 0.449±0.084b | 1.049±0.236b | 13.652±9.796a | 6.589±2.383ab |
| *Eurotiomycetes* | | 5.010±1.398ab | 0.934±0.282c | 6.990±2.442a | 3.269±1.980bc |
| *Agaricomycetes* | | 1.734±1.312b | 1.302±0.901b | 10.994±8.348a | 1.211±0.938b |
| *Ascomycota_unidentified* | | 2.217±1.688b | 0.321±0.044b | 9.609±5.920a | 2.217±0.302b |
| *Rhizophlyctidomycetes* | | 3.676±4.757a | 0.327±0.236a | 0.178±0.081 a | 0.440±0.440a |
| Others | | 2.929±1.390a | 3.176±1.835a | 1.766±1.852a | 1.737±1.135a |
