# Supplementary figures and images for: Changes in soil fungal communities after onset of wheat yellow mosaic virus disease
Source: Front Bioeng Biotechnol. 2022 Oct 17;10:1033991. doi: 10.3389/fbioe.2022.1033991 (PMC9621598; doi:10.3389/fbioe.2022.1033991)

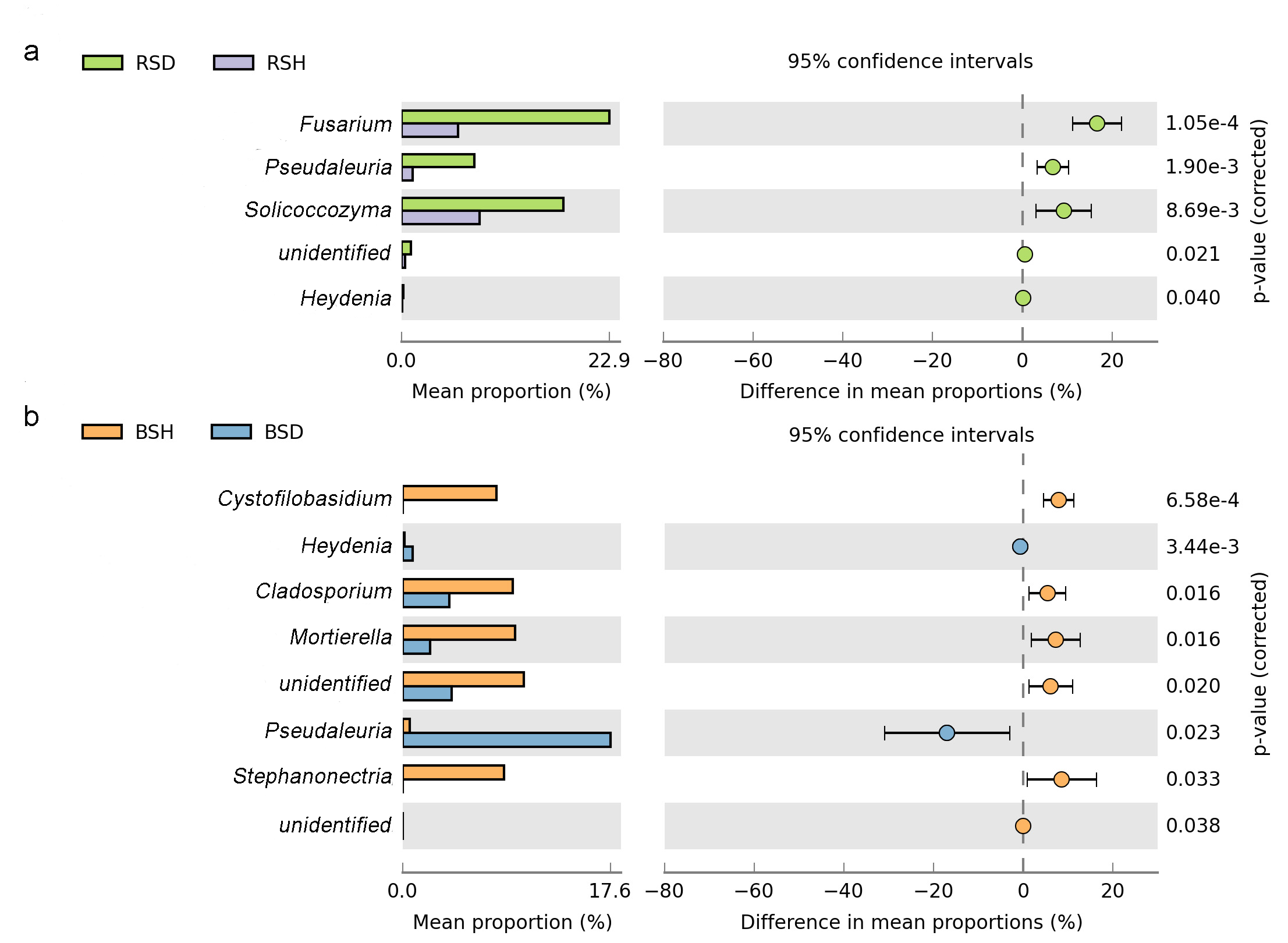

Supplement: Supplementary file 2 [file Image1.TIF]
